# Differential diagnosis between maladaptive daydreaming and ADHD: Immersive daydreaming is not simply inattention

**DOI:** 10.1016/j.ijchp.2025.100616

**Published:** 2025-08-23

**Authors:** Nitzan Theodor-Katz, Nirit Soffer-Dudek

**Affiliations:** Department of Psychology, Ben-Gurion University of the Negev, Beer-Sheva 8410501, Israel

**Keywords:** Maladaptive daydreaming, ADHD, Daydreaming, Nosology, Mind wandering

## Abstract

Maladaptive daydreaming (MD), a syndrome considered by dissociation researchers to represent a dissociative disorder, entails excessive, addictive immersion into narrative and emotional fantasies, impairing functioning and increasing distress. People with MD often meet the criteria for attention-deficit/hyperactivity disorder (ADHD), as addictive and immersive daydreaming causes inattention. Conversely, most people with ADHD do not suffer from MD, yet commonly score highly on the MD self-report screener, questioning the reliability of MD and ADHD symptom checklists. We examined whether assessing a mental pattern of immersive daydreaming improves the reliability of MD classification. A sample of 156 adults comprising four groups: ADHD (*n* = 38), MD (*n* = 49), Both (*n* = 34), and Controls (*n* = 35), underwent clinical interviews and completed self-report scales assessing symptoms and immersive daydreaming. As hypothesized, the MD self-report screener was compromised in the face of ADHD. Immersive daydreaming self-reports counteracted that by adding significant unique predictive value for MD identification in the context of ADHD. This indicates that immersive daydreams are distinct mentation not necessarily characterizing ADHD. We suggest a practical cutoff score for identifying high immersive daydreaming which complements MD screening, improving correct MD identification in the context of ADHD.

## Introduction

Maladaptive daydreaming (MD) is a suggested syndrome that entails recurrent episodes of excessive and prolonged absorption into fanciful daydreams, enjoyable in the short term but leading to significant clinical distress and/or functional impairment (Somer, 2002). The daydreams involve scenarios that are unlikely to occur in real life, whether they are based on real people in fantasized scenarios or fictional characters. These daydreaming episodes could be considered a self-hypnotic process as they are often initiated using music and repetitive movements to facilitate a dissociative state, whereby they are experienced vigorously to the point of detachment from ongoing external reality (Somer, 2023, 2024). Indeed, MD has recently been recognized by dissociation researchers as a condition that falls under the category of dissociative disorders, representing the pathological end of absorption (Soffer-Dudek et al., 2025). MD is typically characterized by a profound yearning to be immersed in a daydream rather than engage in real-life occupations, and by an emotional attachment to the daydream’s characters, as they provide emotional escape and compensation (Somer, Lehrfeld, et al., 2016, Somer, Somer & Jopp, 2016). The act of immersive daydreaming in itself is not inherently pathological and can be an enjoyable pastime for those with high imaginative abilities. However, in MD, the temporary joy experienced by the fantasies may quickly turn into shame about their frequency and time-consuming nature. They may also lead to loneliness as individuals try to conceal them and/or choose their inner world over their actual one, dampening actual social relationships (Chefetz et al., 2023). Additionally, due to the allocation of attention resources towards daydreaming, MD may also cause diminished achievements in work or studies or impair one’s ability to focus on tasks and get things done. Accordingly, MD represents a pathological end of a normative daydreaming spectrum, whereby daydreaming becomes a maladaptive, inflexible, recurring mental pattern, leading to detachment (Soffer-Dudek & Somer, 2022).

MD is not yet formally acknowledged in psychiatric diagnostic manuals, although its formal recognition has been recently advocated for (Soffer-Dudek et al., 2025). Such recognition has been encouraged by several hundreds of sufferers with lived experience of MD, who had been feeling misunderstood by other psychiatric conceptualizations and thus individually reached out to researchers once they discovered the concept of MD, requesting that they continue studying the phenomenon (Bershtling & Somer, 2018). The resulting proliferation of research on MD in the past decade underscores the importance of better understanding this condition and its similarities or differences from existing psychopathological constructs. In 2016, the concept was operationalized with the development of a quantitative self-report scale, the Maladaptive daydreaming scale (Somer, Lehrfeld, et al., 2016), which turned into the MDS-16 when two items were later added (Somer, Soffer-Dudek, Ross & Halpern, 2017). The MDS-16 assesses the yearning to become engrossed in daydreaming activity, impairment in various life domains caused by excessive daydreaming, involvement of kinesthetic elements, and the role of music. This measure became the widespread and consensual measuring tool in this field of research and has subsequently been translated and validated across several languages with adjusted screening cutoffs ranging from 25 to 63 (Abu-Rayya et al., 2019; Balashevych et al., 2024; Balestra, 2019; Breuer, 2022; Catelan et al., 2023; Jopp et al., 2019; Metin et al., 2022; Pietkiewicz et al., 2023b; Schimmenti et al., 2020). A cutoff of 40 has been reported for the English version (Soffer-Dudek, 2025). MD also has suggested DSM-like diagnostic criteria and a compatible structured clinical interview SCIMD (Somer, Soffer-Dudek, Ross & Halpern, 2017).

Research has shown that people with MD are highly distressed and dysfunctional, with high rates of suicidality (Soffer-Dudek & Oh, 2024; Soffer-Dudek & Somer, 2018). Studies that carried out clinical interviews discovered that people with MD also exhibit additional, comorbid psychopathology symptoms, such as depression, anxiety, dissociation, autism spectrum disorder, obsessive-compulsive spectrum disorders, and narcissism (Pietkiewicz et al., 2023a; Renzi & Mariani, 2025; Ross et al., 2020; Somer, Soffer-Dudek & Ross, 2017; West et al., 2023). Although MD has several dissociative properties such as experiential disconnectedness and self-incoherence, no current dissociative disorder can account for the specific symptoms of MD (Soffer-Dudek et al., 2025). Similarly, MD may share common mechanisms with obsessive-compulsive spectrum disorders such as compulsivity (Salomon-Small et al., 2021), and with autism, such as repetitive interests (West et al., 2023), however, again, these disorders are not perfectly overlapping with MD. Although many individuals suffering from MD have significant narcissistic traits (Ghinassi et al., 2023), there is no overt symptom overlap. Notably, a disorder that does share significant symptom overlap with MD is ADHD.

One study, conducting full psychiatric assessments (Somer, Soffer-Dudek & Ross, 2017), found that people with MD tended to meet criteria for several DSM disorders, such as depression, anxiety, and obsessive-compulsive spectrum disorders. However, more than any of the other comorbidities, people with MD most frequently met the diagnostic criteria for attention-deficit/hyperactivity disorder (ADHD), spanning almost 77 % of the sample, the vast majority of whom manifested the inattentive-only subtype. Yet, importantly, many of them explained that their inattention was a secondary result of their addiction to daydreaming. This implies that rather than a true comorbidity, the high rates of ADHD may be misleading as the clinical picture might be better explained by MD. In other words, if MD were included in the DSM, the diagnosis of ADHD would become superfluous in some cases where MD would better explain the symptoms. Indeed, many people with MD typically report that existing diagnostic labels are unhelpful for them (Bigelsen et al., 2016).

Still, the extremely high rates of ADHD in an MD sample could raise concerns as to the differentiation of the two conditions: if the people diagnosed with these two conditions significantly overlap, that would question the necessity of the newer concept of MD. This prompted a complementary study in a sample of people with ADHD, where the authors hypothesized that they would find a much lower incidence of MD (Theodor‐Katz et al., 2022). Finding such a lower incidence would imply that the concepts do not overlap. Indeed, structured clinical interviews identified that only a fifth of the ADHD sample met the criteria for MD, suggesting that although MD rates in the ADHD sample were probably higher than in the general population, most people with ADHD do not suffer from MD, but rather MD represents a separate condition not explained by ADHD symptoms. That study also showed, through self-report, that the MDS-16 was positively associated with ADHD, suggesting that the MDS-16 may be measuring inattention symptoms to some extent. Although they are a relevant part of the MD clinical picture, they may be less specific and thus may misidentify people with ADHD, increasing false positives when screening for MD and thus inflating suspected MD rates, especially in an ADHD population.

Due to the existence of MD and ADHD comorbidity along with consistent reports of positive significant correlations between MD, ADHD, and MW (Catelan et al., 2023; Jopp et al., 2019; Metin et al., 2022; Theodor‐Katz et al., 2022; Theodor-Katz & Soffer-Dudek, 2025), developing a conceptualization of daydreaming that is distinct from other attention impairing thought patterns is needed, as well as outlining the differentiation between ADHD and MD. Evidently, individuals with ADHD tend to struggle to complete a task before starting another one, feel overwhelmed when facing multiple tasks, and experience mental effort as a “lost battle”. These challenges can be attributed to deficiencies in executive functions (Antshel et al., 2014) and they are not typical to MD. People with MD do not often report flight of thoughts (Asherson, 2005), difficulties with meticulous work, difficulties in following instructions, or changing the topic of a conversation often, as typically occurs in ADHD (American Psychiatric Association, 2022). The main characterization of MD is atypical and intense daydream engagement with a high degree of focus and cognitive and affective involvement in fanciful storylines that are unlikely to occur in real life (Theodor-Katz & Soffer-Dudek, 2025).

Vivid and absorbing fantasies, not necessarily pathological in and of themselves, have been labeled as “immersive daydreaming” (West & Somer, 2020). However, this distinctive terminology is quite recent, and this perspective had not been present in the research field of daydreaming in previous decades. Several studies have relied on murky definitions and blurred the boundaries between different mental states. For example, the Imaginal Processes Inventory (Giambra, 1980) refers to both fantasized and realistic content as daydreaming. Similarly, the research field of sluggish cognitive tempo, later referred to as cognitive disengagement syndrome, a closely related syndrome to ADHD, is also characterized by inattention and daydreaming that are interchangeably considered as MW (Becker, 2021; Becker & Barkley, 2021). Recently, several authors have pointed to the need to better conceptualize the differences between immersive daydreaming and unfocused mentation or MW (Lawson & Thompson, 2024; Shimoni & Axelrod, 2024; West & Somer, 2020).

This need is especially pressing following the recent identification of MD, which causes distress and necessitates the development of targeted interventions, based on a thorough understanding of the thought patterns enveloping the client’s mind. MD includes immersive qualities (i.e., feeling extremely involved in the daydream) and dissociative ones (Soffer-Dudek & Somer, 2022). Specifically, an empirical dissociative factor labeled as “absorption and imaginative involvement” represents a state of narrowed attention and total immersion in a single stimulus to the point of obliviousness to one’s surroundings (Carlson & Putnam, 1993); Accordingly, immersion in MD reduces awareness to the external environment in favor of an inner world that is often reported to feel more “real” than real life (Soffer-Dudek & Somer, 2022). A Factor analysis showed that dissociative absorption was distinct from ADHD and MW, whereas the latter two could not be reliably separated (Soffer‐Dudek, 2019). This supports the idea that the syndrome of MD and its associated mental pattern of immersive daydreaming are distinguished from the syndrome of ADHD and its associated mental pattern of MW. Indeed, MD is predicted by dissociation in general, and absorption in particular (Ferrante et al., 2022; Somer & Herscu, 2017) and has been conceptualized as a pathological form of dissociative absorption (Soffer-Dudek & Somer, 2022). Consequently, it is reasonable to conclude that MD is not a phenotype or a result of ADHD. We suggest that although MD and ADHD may both manifest as distraction and having trouble focusing on tasks, they emanate from distinct mental patterns: while the first is characterized by immersive daydreaming, the second is prototypically characterized by MW.

Given the gap in the current formulation and measurement of different distraction types, specifically immersive daydreams characterizing MD versus MW characterizing ADHD, the present study aimed to: (a) examine whether the presence of ADHD reduces the diagnostic accuracy of MDS-16 screening for MD; and (b) improve MD screening by better distinguishing it from ADHD. To accomplish this goal, we used a recently developed self-report scale assessing immersive daydreaming. The Daydreaming Characteristics Questionnaire (DCQ; (Theodor-Katz & Soffer-Dudek, 2025)) was created to gauge the distinguished structure and content of thoughts characteristic of MD rather than ADHD or MW. We conducted detailed clinical interviews and used them as a predicted criterion to examine the usefulness of self-report measures for screening. Our hypotheses were:

1. The MDS-16 (with its previously reported cutoff of 40) would identify MD cases well when ADHD cases are not present in the sample, replicating previous research that did not specifically target ADHD.
2. The MDS-16 would demonstrate diminished performance in its ability to identify MD in the full sample, which encompasses many people classified with a dominant presentation of ADHD symptoms. Specifically, the MDS-16 cutoff score of 40 will tend to produce false positives.
3. Adding the new DCQ as a second predictor, complementing the MDS-16, will significantly improve the classification of MD in the full sample (that includes ADHD cases).

## Materials & methods

### Participants and procedure

The study was approved by the university’s human subjects research committee in accordance with the provisions of the World Medical Association Declaration of Helsinki, approval # 2228–1. Participants were recruited through social media platforms according to the study target populations: designated forums for MD, ADHD, and forums concerning general interest in psychology or research participation. Participants who enrolled for the study via MD/ ADHD forums were compensated with personal feedback on their reported symptoms and took part in a raffle for a $100 gift card. Participants who enrolled via general platforms were compensated with a $25 gift card as a motivator for participation. Notably, however, final study group allocation was determined through the clinical interviews, rather than the recruitment context. All participants signed an informed consent form prior to their participation. Participants were assessed with two structured clinical interview protocols, for MD and ADHD symptoms, administered online via video conference technology. Before the interview, they also completed a battery of online self-report questionnaires, and after the interview they completed a momentary sampling procedure outside of the scope of the current investigation. Inclusion criteria required completing all three study stages. Participants were excluded from the study when there were inconsistencies in the reported location data compared to the IP detected by the platform, in cases of significantly short time duration for questionnaire completion, or in cases of vague contradicting answers throughout the interview suggesting ingenuine participation due to the monetary compensation offered to the participants. Recruitment continued until all groups (see below) had a minimum of *n* = 30, resulting in a final sample size of *N* = 156. Following the clinical interviews, participants were classified into four groups: MD (*n* = 49), ADHD (*n* = 38), a group with co-morbid MD and ADHD (Both, *n* = 34), and a group with neither (Control, *n* = 35). The interviews lasted 75–120 min and were carried out predominantly by the first author, who is a licensed clinical social worker, and a subset were carried out by a psychology student who underwent targeted training by the first author. Participants were advised to turn to their health provider in case of distress and were also invited to contact the researchers if they felt distressed following the study. Hotline websites and phone numbers for mental health support were also provided.

Table 1 presents sample characteristics. As can be seen in the table, the MD and the Both groups were significantly younger (∼30y) than the ADHD group (∼40y), and the MD group was also significantly younger than the control group (∼37y). The table also shows that all groups consisted predominantly of females (∼60 % - 80 %), with no significant differences in proportions between groups (*χ^2^* = 5.05, *df* = 6, *ns*). The majority of the participants in all groups reported that their income level was moderate (71.05 % in ADHD and 53.06 %−59.00 % in the other three groups). Unemployment seemed to be more prevalent in the MD (34.70 %) and Both (38.23 %) groups compared to the control (25.71 %) and ADHD (28.95 %) groups. Most individuals were from North America (59 %), a fifth (20.5 %) from Europe, 10.9 % from Asia, 4.5 % from South America, 3.2 % from Africa, and 1.9 % from Oceania. Finally, Table 1 also presents participants’ self-reports of either past or present clinical diagnoses, suggesting high comorbidity rates in the clinical groups, with depression and general anxiety disorder being the most prevalent reported diagnoses. Three participants reported psychotic disorders, however, due to no indication of active psychosis during the interview they were not excluded from the study.

**Table 1:** Descriptive statistics of demographics and participants’ self-reported psychiatric diagnoses.

	MD*_n_*_=__49_	ADHD*_n_*_=__38_	Both*_n_*_=__34_	Control*_n_*_=__35_
**Age**†				
M (SD)	29.75 (9.85)	40.13 (15.8)	30.90 (9.89)	37.11 (11.72)
Range	18–55	19–76	20–68	18–71
**Gender**				
Female	65.30 % (32)	71.05 % (27)	79.41 % (27)	60.00 % (21)
Male	28.57 % (14)	23.68 % (9)	14.70 % (5)	37.14 % (13)
Other	6.12 % (3)	5.26 % (2)	5.88 % (2)	2.85 % (1)
**Education level**				
Secondary or lower	17.14 % (6)	20.40 % (10)	18.42 % (7)	26.47 % (9)
Post-secondary or Bachelor's degree	40 % (14)	61.22 % (30)	50 % (19)	55.88 % (19)
Graduate studies	42.85 % (15)	18.36 % (9)	31.57 % (12)	17.64 % (6)
**Self-reported past or present clinical diagnoses % (*n*)**				
Depression	40.81 % (20)	34.21 % (13)	35.29 % (12)	17.14 % (6)
General anxiety disorder	32.65 % (16)	26.31 % (10)	38.23 % (13)	14.28 % (5)
Obsessive-compulsive disorder	10.20 % (5)	7.89 % (3)	8.82 % (3)	0.0 % (0)
Social anxiety disorder	14.28 % (7)	7.89 % (3)	8.82 % (3)	2.85 % (1)
Dissociative disorders	6.12 % (3)	5.26 % (2)	5.88 % (2)	0.0 % (0)
Post-traumatic stress disorder	8.16 % (4)	7.89 % (3)	20.58 % (7)	5.71 % (2)
Autistic spectrum disorders	6.12 % (3)	0.0 % (0)	2.94 % (1)	2.85 % (1)
Substance abuse disorder	4.08 % (2)	2.63 % (1)	2.94 % (1)	0.0 % (0)
Psychotic disorders	2.04 % (1)	5.26 % (2)	0.0 % (0)	0.0 % (0)
Bipolar disorders	4.08 % (2)	7.89 % (3)	8.82 % (7)	0.0 % (0)

*Note:* Data is presented in percentages. In brackets are the absolute numbers.

† Planned contrasts analysis for differences in age means among groups: Control; MD 7.36, 95 % *SE* 2.47, *CI* [0.94,13.79], *t*(149)=2.98, *p* = .017, Control; ADHD −3.02, 95 % *SE* 3.31, *CI* [−11.63,5.59], *t* (149)=−0.91, *p* = .079, Control; Both 6.21, 95 % *SE* 2.67, *CI* [−0.71,13.13], *t* (149)=2.32, *p* = .09*6*, MD; ADHD −10.39, 95 % *SE* 3.00, *CI* [−18.18,−2.59], *t* (149)=−3.46, *p* = .003, MD; Both −1.16, 95 % *SE* 2.26, *CI* [−7.04,4.73], *t* (149)=−0.51, *p* = .095, ADHD; Both 9.23, 95 % *SE* 3.16, *CI* [1.01,17.44], *t* (149)=2.91, *p* = .021.

### Measures

#### Structured clinical interviews

##### Maladaptive daydreaming

We administered a Structured Clinical Interview for MD (SCIMD; (Somer, Soffer-Dudek, Ross & Halpern, 2017)). The interview starts with an explanation delineating what would count as a daydream as compared to general distractions (e.g., remembering one’s to-do list). Four diagnostic criteria are then evaluated: (A) the presence of vivid, persistent, recurring daydreams; (B) the absorptive nature and the constant yearning to daydream; (C) possible distress caused by daydreaming activity (e.g., shame or embarrassment, low self-worth due to experiencing a gap between ideal fantasized self and real self, feeling guilty due to the wasted time) and functional impairment associated with the compulsive daydreaming (e.g., less intimate relationships, reduced concentration, underemployment); and (D) differential diagnosis. Specifically, we asked about symptoms of ADHD, substance abuse, psychotic disorders, obsessive-compulsive disorder, dissociative identity disorder, and post-traumatic stress disorder. The interviewers added follow-up questions in cases of vague answers***.*** For MD classification participants required a positive answer for criterion A, at least one symptom of criterion B, either distress or at least one area of functioning negatively affected (criterion C), and having no other mental or physical conditions better explaining their daydreaming’s negative effect (criterion D). If the participant also met the classification for the ADHD group, where the ADHD clinical presentation could not explain the MD-related clinical picture and vice versa, they were classified to the Both group.

##### Attention-deficit/hyperactivity disorder (ADHD)

ADHD was assessed using the Diagnostic Interview for ADHD in Adults (3rd edition) DIVA-5 (Kooij et al., 2019). The DIVA-5 asks about the core symptoms required for a DSM-5-based ADHD diagnosis. The interview included four parts; a short explanation of its aims and length, followed by a list of 18 questions evaluating each of the 18 symptom criteria of ADHD regarding both childhood and adulthood. Participants were given examples of the typical impairments commonly associated with each symptom. Next, interviewees were asked to indicate if they always experienced such difficulties, specifically before the age of 12. Lastly, the interview inquiries about the manifestation of symptoms in work, education, relationships and familial life, social contacts, free time, and self-image, also both in childhood and adulthood. Participants were assigned to the ADHD group if they reported at least five symptoms of inattention/ hyperactivity and impulsivity during adulthood and three compatible symptoms starting before age 12, and if the symptoms generated functional impairment in at least two areas during adulthood and childhood. As before, if a participant also met diagnostic criteria for MD (not better explained by their ADHD symptoms), they were assigned to the Both group.

#### Self-report questionnaires

##### Immersive daydreaming

The Daydreaming Characteristics Questionnaire (DCQ) was developed to assess the characteristics of immersive daydreaming as a unique thought pattern, differentiated from general MW which characterizes typical ADHD (Theodor-Katz & Soffer-Dudek, 2025). It consists of one categorical item directly asking about the nature of the person’s most dominant distraction (worrying, planning future tasks, fantasizing about imaginative events, gazing blankly, or “other”), and an additional six continuous items rated on a 5-point Likert scale, representing one immersive daydreaming (IDD) latent factor. IDD items explore the following dimensions: fantasized versus realistic content, repetitive versus diverse themes, evolving narrative versus a fragmented singular scenario, vividness, emotional range during daydreaming episodes, and positive valence. The final score is calculated by a separate coding for the categorical question and the mean score for IDD. Cronbach’s alpha for IDD was α = 0.83.

##### Maladaptive daydreaming

MD was assessed using the 16-item Maladaptive Daydreaming Scale (MDS-16), the most widely-used measure in MD research, which is rated on an 11-point Likert scale (Somer, Lehrfeld, et al., 2016; Somer, Soffer-Dudek, Ross & Halpern, 2017). The MDS-16 items include yearning to become absorbed in daydreams, daydreaming-generated impairment in functioning or psychological distress, and daydream-related use of movement or music. The English version of the MDS-16 has an empirically derived cut-off score of 40 suggesting elevated risk for MD (Soffer-Dudek, 2025). Cronbach’s alpha was α = 0.93.

##### Attention-deficit/hyperactivity symptoms

ADHD symptoms were assessed using the Barkley Adult ADHD Rating Scale-IV (BAARS-IV; (Barkley, 2011). The BAARS-IV is aimed at evaluating ADHD symptoms in daily life. We used the BAARS-IV long form for adults’ current symptoms, which is a self-report questionnaire consisting of four subscales assessing inattention, hyperactivity, impulsivity, total ADHD level (based on the latter three subscales), and sluggish cognitive tempo, which was not part of the focus of the present study because of its ambiguous definition regarding daydreaming as a symptom. Items are rated between 1 (Never or rarely) to 4 (Very often), and final scores are calculated by determining how deviant the respondent is on the scale relative to the normal population, according to their age (Barkley, 2011). Cronbach’s alpha was 0.93 for Inattention, 0.83 for both hyperactivity and impulsivity, and 0.91 for total ADHD.

##### Mind wandering

MW was assessed using the Mind Excessively Wandering Scale (MEW) (Mowlem et al., 2019). The MEW was developed to assess excessive MW as a co-occurring aspect of ADHD among adults with ADHD. The scale comprises 12 items rated on a 4-point Likert scale, ranging between 0 (not at all or rarely) and 3 (nearly all of the time), for example, “I find it difficult to think about one thing without another thought entering my mind”. Cronbach’s alpha in the present study was 0.93.

### Statistical analysis

First, we present partial correlation coefficients on the full sample between self-report study variables, controlling for age, which was uneven across groups. To support our contention that the existing MDS-16 cannot accurately classify MD versus controls when ADHD is present in some of the participants, we evaluated the performance of the previously suggested cutoff score (Soffer-Dudek & Somer, 2022; Somer, Soffer-Dudek, Ross & Halpern, 2017) using a Receiver operating characteristics (ROC) analysis (Janssens & Martens, 2020). ROC analysis illustrates a set of different thresholds and their tradeoff between sensitivity (the percent of correctly identified positive cases) and 1 - specificity (the percent of correctly identified negative cases). The Area under the curve (AUC) metric represents the overall performance of the scale. First, to test for the replicability of the clinical cutoff findings of (Somer, Soffer-Dudek, Ross & Halpern, 2017) who compared people with MD to matched controls, we analyzed only control and MD participants, excluding all ADHD cases. Next, we conducted two more ROC analyses; one to assess the MDS-16′s discriminating performance over the entire sample (all four groups, divided into MD versus non-MD), and another one focusing only on participants with ADHD, either with or without comorbid MD. Participants who were assigned either to the MD group or the Both group were classified as positive for MD while the ADHD and control groups were classified as negative. We hypothesized that differentiation using the MDS-16 would be much less accurate in the presence of ADHD cases. After examining the MDS-16 performance, we examined whether either of the DCQ elements (categorical item and IDD) had a significant unique contribution over and above the existing MDS-16 in predicting a person’s probability for MD using two respective logistic regression models. Then, we performed another ROC analysis to extract a cutoff for the DCQ’s continuous IDD factor, to contribute practically to MD identification using self-report. Finally, we examined whether the DCQ had a significant predictive value for MD over and above the MDS-16 score. Accordingly, two logistic regression models were applied; in the first model the IDD and the MDS-16 scores were inserted as the predictors and MD as the outcome variable. In the second model the single categorial item and the MDS-16 score served as predictors and MD as an outcome.^1^

### Transparency and openness

Research components, including the dataset and analyses code, are available on the Open Science Framework (OSF) at [https://osf.io/7ybkf/?view_only=787f0323dded41f99e8f7d926bedbb87]. Since the Barkley Adult ADHD Rating Scale-IV BAARS-IV;(Barkley, 2011) and the Diagnostic Interview for ADHD in Adults (3rd edition) DIVA-5 (Kooij et al., 2019) are subject to copyright restrictions, they and their scoring are not shared.

## Results

### Correlations between self-report scales

First, we present means and standard deviations for continuous, self-reported study variables, as well as partial correlations controlling for age. As can be seen in Table 2, the mean MDS-16 score of the ADHD group was quite high (*M* = 34.87) compared to the control group (*M* *=* 19.13), pointing to the compromised differentiation we hypothesized regarding self-reported MD and ADHD. Also in accordance with our expectations, the association between IDD and MD (*r* = 0.71) seemed to be much more robust than IDD’s association with MW, ADHD, and ADHD inattention presentation (*r* = 0.31, *r* = 0.23, *r* = 0.30, respectively), supporting the idea of immersive daydreaming as a specific characteristic of MD. Accordingly, comparing the IDD mean scores using planned contrasts ANOVA, indicated that the IDD mean score of all participants with MD (MD and Both) were significantly higher than the ones of non-MD participants (ADHD and control) (difference = 1.09, 95 % CI [0.81, 1.37], t (147)=7.62, *p* < .001), whereas no significant differences emerged within those groups (MD vs. Both: difference = 0.13, 95 % CI [−0.23, 0.50], t (147)=0.72, *p* = .47; controls vs. ADHD: difference =0.19, 95 % CI [−0.19, 0.58], t (147)=0.99, *p* = .32 0.001) .^2^ These results strengthen the idea that IDD taps onto a specific aspect of MD which is less confounded with ADHD.

**Table 2:** Descriptive statistics and partial pearson correlations between study variables, controlling for age.

	MD	IDD	Inattention	Hyper-activity	Impuls-ivity	ADHD	MW
IDD	.714^⁎⁎⁎^						
Inattention	.484^⁎⁎⁎^	.307^⁎⁎⁎^					
Hyperactivity	.200*	.128	.492^⁎⁎⁎^				
Impulsivity	.105	−0.010	.469^⁎⁎⁎^	.506^⁎⁎⁎^			
ADHD	.391^⁎⁎⁎^	.227*	.899^⁎⁎⁎^	.762^⁎⁎⁎^	.733^⁎⁎⁎^		
MW	.491^⁎⁎⁎^	.310^⁎⁎⁎^	.692^⁎⁎⁎^	.507^⁎⁎⁎^	.422^⁎⁎⁎^	.702^⁎⁎⁎^	
Control- *M (SD)*	19.13 (14.86)	1.61 (0.80)	1.68 (3.72)	1.68 (2.42)	1.66 (1.93)	30.02 (6.87)	12.50 (7.17)
MD- *M* *(SD)*	64.55 (12.74)	2.90 (0.75)	2.59 (4.90)	2.18 (3.30)	1.81 (2.68)	41.53 (8.32)	22.12 (8.13)
ADHD- *M (SD)*	34.87 (19.91)	1.86 (1.02)	2.71 (5.85)	2.26 (3.88)	2.28 (3.44)	44.90 (9.81)	20.84 (8.61)
Both- *M* *(SD)*	57.58 (18.95)	2.75 (0.74)	3.10 (4.92)	2.42 (3.28)	2.52 (3.05)	50.20 (8.71)	25.66 (6.98)
All- *M* *(SD)*	45.78 (24.47)	2.32 (0.99)	2.52 (6.60)	2.14 (3.50)	2.05 (3.11)	41.64 (10.96)	20.39 (9.00)

*Note:* Correlations are presented for the entire sample controlling for age. MD = maladaptive daydreaming using the MDS-16, IDD = Immersive daydreaming, Inattention = ADHD inattention presentation using the BAARS-IV, Hyperactivity = ADHD hyperactivity presentation using the BAARS-IV, Impulsivity = ADHD impulsivity presentation using the BAARS-IV, ADHD = ADHD total score using the BAARS-IV, MW = mind wandering using the MEW*.*

*p < .05, ***p < .001 levels*.* Non-significant correlations are presented in gray font. *M* = mean score, *SD* = Standard deviation*.*

### ROC analysis for the MDS-16

**Fig. 1:**
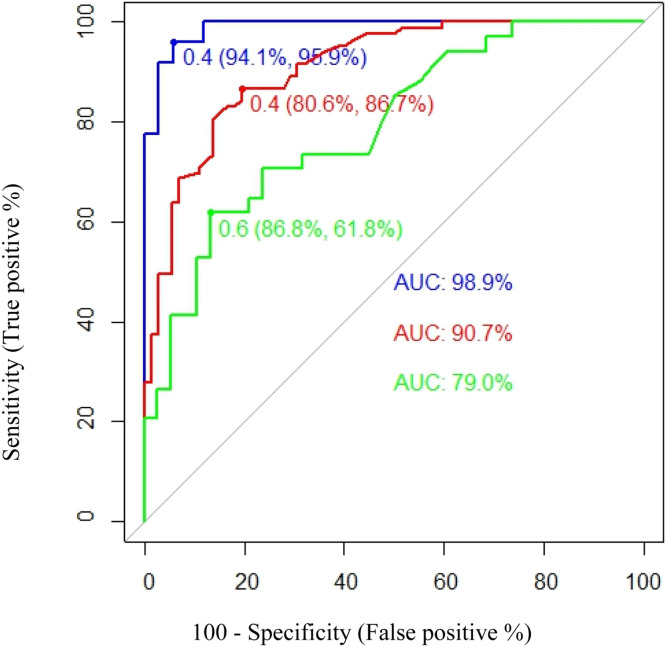
Receiver operating characteristics (ROC) analysis for the MDS-16.

*Note:* The blue line represents a ROC analysis in a sample of MD and Control groups only (i.e., excluding anyone with ADHD, regardless of their MD status). The red line represents a ROC analysis in the entire sample (MD/Both versus Control/ADHD-only). The green line represents a ROC analysis for only ADHD groups (Both versus ADHD-only). AUC (area under the curve) percentiles represent the probability of correctly identified persons.

**Fig. 2:**
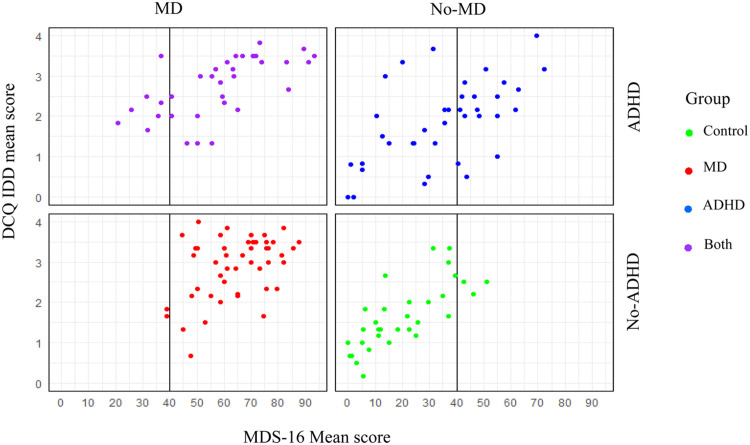
Dispersion of Scores on the MDS-16 and DCQ-IDD.

*Note:* The figure depicts the dispersion of the MDS-16 and DCQ-IDD scores for each of the interview-classified groups in the study: co-morbid ADHD and MD (top left, purple dots), ADHD only (top right, blue dots), MD only (bottom left, red dots) and controls (bottom right, green dots). The vertical line in each of the four graphs represents a score of 40 on the MDS-16, which is the previously known cutoff score for suspected maladaptive daydreaming (MD). DCQ = Daydreaming characteristics questionnaire, IDD = Immersive daydreaming factor.

As can be seen in Fig. 1, when applied to a sample of participants with MD and control classification only (i.e., excluding the two groups with ADHD), the previously identified MDS-16 cutoff score of 40 performed nearly perfectly (AUC = 98.9 %, *CI* [0.97, 1.00]). Sensitivity was 96 % and specificity 94.1 %. However, when applying the ROC analysis over the entire sample (comparing negatives: control/ADHD, to positives: MD/Both), sensitivity decreased to 86.7 % and specificity to 80.6 %. The results indicated almost 20 % false MD classification with an AUC of 90.7 %, *CI* [0.86, 0.95]. Examination of the differences between the AUC of only the MD and Control groups compared to the entire sample using DeLong test (DeLong et al., 1988): *D* *=* *3.33, df = 188.66, p = .001*, indicated a significantly lower AUC when ADHD was included in the sample. Moreover, when focusing on an ADHD sample and attempting to distinguish ADHD-only versus Both, the AUC was even further reduced (79.0 %, *CI* [0.68, 0.89]) and the scale’s accuracy further deteriorated with a sensitivity of 73.52 % and specificity of only 44.73 %. A significant difference was also detected between the total sample AUC and the ADHD and the Both groups (*D = −2.03, df = 98.89, p = .04*), suggesting the latter has a significantly smaller probability to detect MD accurately. Fig. 2 presents the dispersion of cases above and below the existing MDS-16 cutoff score in each of the four groups. The figure illustrates clearly the excellent performance of the cutoff score among groups with no ADHD. However, as can also be seen in the figure, the ADHD-only group presented an inconsistent pattern of MDS-16 score distribution with 47.46 % of individuals scoring over the cutoff, adding many false positives to the sample.^3^ Interestingly, the Both group had only 80 % of its participants reaching the cutoff, suggesting too many false negatives as well. We attempted to examine the usefulness of a more rigorous MDS-16 cutoff for identifying MD among ADHD cases, but we were unable to detect a cutoff that would provide both adequate sensitivity and specificity. Thus, increasing the MDS-16 cutoff does not seem like an appropriate solution for detecting cases of MD in a sample with ADHD, underscoring the need for the addition of a different measure specifically aiming to differentiate ADHD from MD, such as the DCQ.

### Predicting a maladaptive daydreaming diagnosis with the addition of the DCQ

Next, we wished to examine whether the addition of the DCQ can enhance the correct identification of MD in the presence of ADHD. We thus conducted two logistic regression models, predicting the probability of being clinically classified with MD using two predictors: the MDS-16 and a score from the DCQ. The categorical item (dummy variable) and IDD (continuous predictor) were included in separate models because of their shared variance. In each model, we examined whether the DCQ element had any incremental value in predicting MD over and above the existing MDS-16. Both the IDD factor and the categorical item were significant unique predictors of MD with a contribution beyond the effect of the existing MDS-16 cutoff (MDS-16 alone: AIC = 155.09; MDS-16 and categorical item: AIC = 138.19; MDS-16 and IDD: AIC = 143.25). Specifically, examination of the model with the categorical item indicated that even just asking directly about one’s distracting thought content using a straightforward single item was useful; it significantly increased the probability of belonging to the MD group when people reported mostly being occupied with fantastical daydreams (log *OR* = 2.41, *OR* = 3.53, *CI*[1.51, 7.63], *z* = 2.90, *p* = .003). The model including the IDD factor indicated that IDD predicted an increased probability for MD as well (log *OR* = 0.91, *OR* = 2.48, 95 % *CI* [1.52, 4.20], *z* = 3.53, *p* < .001), such that an increase of one unit on IDD was on average associated with an increase of 12.9 percentage points in their likelihood to suffer from MD (95 % CI [6.54, 19.2]). A person with an IDD score of 0 had on average a probability of 0.12 to suffer from MD, while a person with an IDD score of 4 had, on average, a much higher probability of 0.84.

The regression models suggested that DCQ elements add significant information and accuracy to MD prediction. Hence, we opted to also identify an optimal cutoff score for the continuous IDD, to enable ease of screening in future studies and clinical settings. We performed another ROC analysis, this time using IDD scores (rather than MDS-16 scores), on the full sample (control/ADHD versus MD/Both). We detected an optimal cutoff score of *M* = 2.27. Notably, the IDD aims to serve as a screening aid for clinical diagnosis by complementing the MDS-16 in the presence of ADHD cases, rather than acting as a stand-alone measure. In our full sample *n* = 73 were classified to the control or ADHD group according to the interview, although *n* = 21 of them exceeded the MDS-16 cutoff. When we added the criterion of passing the new IDD cutoff to the initial criterion of only passing the MDS-16 cutoff, the rate of false positives was reduced to *n* = 11, i.e., the false positive rate dropped by 47.61 %, whereas the identification of true positives was reduced by only 20.27 %. When adding the DCQ's single item to the MDS-16, the false positive rate was only *n* = 6, a 71.42 % reduction of false positive cases. However, the true positive rate was reduced by 37.83 %. These findings suggest that, although its reduction in false positives is accompanied by a certain compromise in the identification of true positives, the DCQ can partly aid in avoiding the mistaken classification of ADHD cases as MD using self-report tools.

## Discussion

In line with H1, and replicating previous research, applying the cutoff of 40 for the MDS-16 in a non-ADHD population identified MD cases near perfectly, supporting the usefulness of that cutoff when inattention is not a predominant complaint. However, in accordance with H2, allowing for cases of ADHD (with and without co-morbid MD) to be part of the sample significantly diminished the accuracy of the MDS-16 as a screener. In particular, the specificity of the cutoff was largely reduced for the ADHD-only group, resulting in high rates of false positive classification of MD, as we had expected. Additionally, sensitivity was compromised, as several participants from the Both group were under the MD cutoff. The reduction of specificity is not surprising, as the MDS-16 screening cutoff was originally validated on a group of individuals with MD and a matched control group, not necessarily suffering from inattention. The rate of MD frequency according to self-report screening has been shown to be inflated among people with ADHD (Theodor‐Katz et al., 2022). A decreased accuracy of the MDS-16 screening cutoff was also observed in a sample of individuals diagnosed with autism spectrum disorder, where 43 % exceeded the cutoff although only a fifth met MD diagnostic criteria according to their interview (West et al., 2023). Possibly, the MDS-16 may exhibit poor specificity whenever used in neurodivergent populations, who may share overlapping symptoms with MD. This underscores the importance of clearly delineating and assessing distinguishing features of different diagnostic entities. As such, the compromised performance we detected of the MDS-16 cutoff in our ADHD sample confirmed the necessity to improve its discrimination capacity in the face of attention difficulties by adding items focusing on immersive daydreaming, which was our goal in this study.

Importantly, our findings showed that the DCQ significantly improved the prediction of MD, both with the simple straightforward item asking about one’s dominant distraction (with fantasies as the chosen answer) and using the continuous IDD scale. Specifically, a high immersive daydreaming score, indicating more vivid, sequential, unrealistic daydreams, characterized by positive emotions and an emotional range, with a repetitive theme, predicted a greater likelihood for MD. Accordingly, when focusing on self-report scales, IDD had a robust correlation (of over 0.70, indicating >50 % shared variance) with the MDS-16 but only a moderate correlation with ADHD, replicating the findings of Theodor-Katz and Soffer-Dudek (2025), but this time on a clinical sample, that underwent interviews to confirm their diagnoses. Notably, the associations were larger in this mixed clinical and control sample, perhaps due to increased variance range compared to a non-clinical population. The specific effectiveness of IDD in predicting MD and differentiating it from ADHD supports the idea that immersive daydreams uniquely differ from other distraction types like MW, can be effectively quantified and assessed, and can aid in the diagnosis of psychopathological conditions. Regarding this idea, we provided a suggested cutoff score of 2.27 for IDD as an addition to the MDS-16 and its existing cutoff. Our findings provide further evidence that ADHD cannot fully explain MD symptoms. The results also showed that adding a single direct question probing one’s predominant distraction is advantageous in predicting MD.

By improving diagnostic classification accuracy, the DCQ promotes the ability to differentiate immersive daydreaming from ADHD distractions. To our knowledge, the present study is the first to separately measure immersive daydreaming and MW and to inquire about their unique association with ADHD and MD in clinical samples. Although the idea that daydreaming and MW differ from one another is not new (Dorsch, 2015; Stawarczyk, 2018), scientific studies on ADHD, MW, and daydreaming continue to use daydreaming and MW scales interchangeably, failing to delineate the boundaries between them (Osborne et al., 2023; Yip et al., 2023; Zhang et al., 2023). By shedding light on the construct of immersive daydreaming, the DCQ carries a potential new understanding of the different mechanisms resulting in compromised attention. Accordingly, MD can be understood as pathological dissociative withdrawal towards immersive daydreams, negatively affecting attention as a result, but not attributed to attentional deficiencies. Conversely, ADHD can be viewed as a pathology characterized by attention deficits leading to disorganized and unstable attention (Liu et al., 2022) in which distractibility is associated with unfocused, rapidly changing thoughts, without a repeated structured pattern (Antshel et al., 2014). Hence, ADHD could be conceptualized as a primary problem of disorganized attention whereas in MD the compromised attention is secondary to the main phenomenology of addictive daydreaming. Facing difficulties in staying present and occupied in real life due to the extreme longing to be fully immersed in self-soothing daydreams, as reported in MD, does not explain the multifaceted structure seen in ADHD where one has a tendency to jump between different tasks leaving them unfinished, feeling overwhelmed in the face of mental effort and avoiding it, being forgetful and disorganized, experiencing difficulties following instructions, and making careless mistakes (Asherson, 2005). Equivalently, from our clinical impression based on the study interviews, such ADHD symptoms are not prominent markers of the inclination to withdraw inwardly for prolonged hours into unrealistic vivid daydreams providing short-term emotional compensation as described by participants with MD symptoms. These results strengthen the idea that MD should be considered as a separate clinical condition in psychiatric manuals (Soffer-Dudek et al., 2025) and that it is not equivalent to ADHD (Theodor‐Katz et al., 2022).

Importantly, when we started out the study, we believed that many cases of MD are wrongly diagnosed as ADHD, and that true comorbidity between them is rare. However, throughout the interviews, it became clear that there were many cases of true ADHD – MD comorbidity (who eventually formed the sizeable Both group). This finding indicates that ADHD isn't necessarily a differential diagnosis in cases of MD, but rather, they can co-exist. The clinical picture that emerged in these cases was often of exacerbated distress following their dual struggle. The MD generated withdrawal from actively pursuing real-life goals in favor of daydreaming, and even when they managed to stop engaging with their daydreams, they experienced ceaseless distracting thoughts of other types. Interestingly, 20.58 % of the Both group did not reach the MDS-16 cutoff, i.e., their MD was undetected by self-report. Further investigation is needed to explore the characteristics of MD-ADHD comorbidity and its unique effect on wellbeing and prognosis. Notably, an alternative conceptualization to comorbidity could be that immersive daydreaming is not a disorder but a transdiagnostic symptom, complicating any existing disorder. We believe that MD is better conceptualized as a disorder due to its complex clinical picture involving a host of additional symptoms associated with the immersive daydreaming (e.g., stereotypical movement, emotional engagement with fantasized content, associated shame and guilt, a fragmented or dual sense of self, and repeated failed attempts at reducing daydreaming). The results of the present study support this conceptualization by showing the differential mental pattern of each syndrome (MD versus ADHD).

Whereas the complexities of symptom cross-influences were meticulously probed in the clinical interview, when relying on self-report the addition of the DCQ was only partially effective in improving MD identification and reducing false MD classification. We assume that there might be additional markers other than immersive daydreaming that may help differentiate MD from ADHD, which were not examined in the present study. Specifically, we surmise that aspects pertaining to *time* may bear significance in explaining the gap between the interviews and the scales, as these aspects may have been probed in the interviews but missed by the self-report scales. This includes: 1. The duration of a daydream episode and any of its properties that may change over the course of a single episode; 2. the accumulated time spent or dedicated to daydreaming activity; and 3. the time of day in which daydreaming most frequently occurs. During the interviews, participants were asked to clarify their reports of prolonged frequent daydreaming activity. Specifically, we probed whether the daydreams they referred to were short-lived/ fragmented or fading and changing rapidly, or whether a daydream was a prolonged experience. Additionally, we asked participants to quantify the frequency of daydreaming engagement (e.g., whether it was a daily or a weekly experience) and the average time for each daydreaming episode (a few seconds, a few minutes, an hour, or longer). Accordingly, it has recently been suggested to amend the clinical criteria for MD with a new criterion based on objective time spent, both throughout the day and during a single episode (Soffer-Dudek et al., 2025).

Additionally, if participants reported daydreaming mostly at night before falling asleep, we inquired whether the reason for daydreaming in that time frame was because daydreaming was a strategy for falling asleep, or whether it was because these were the only times they had in which no one would interrupt them. The former may be a normal and adaptive way of entering the pre-sleep state whereas the latter may indicate a constant yearning and addiction to daydreaming, limited by circumstances. Combining these indications assisted the interviewers in understanding whether the reported daydreams were indeed immersive and addictive. For example, if an interviewee reported that their daydreams last no longer than a few seconds or minutes, albeit happening several times a day, or mostly serving as a means to facilitate sleep onset, they were not classified as positive for criterion A for MD. Future studies may benefit from addressing the time component (diurnal routine and single episode duration) as a possible factor in MD self-report measures.

Several limitations of the present study should be acknowledged. On one hand, the addition of the DCQ significantly and uniquely predicted MD and improved MD classification by reducing the false positive rate by 47.61 % with the IDD and by 71.42 % with the single item, compared to the MDS-16 alone. However, on the other hand, this improvement was far from perfect; correctly identifying MD in an ADHD sample is still a significant challenge. The remaining scientific gap between the clear prototypical clinical picture of MD and its theoretical conceptualization as a dissociative-addictive disorder versus murky diagnostic accuracy when faced with MW and ADHD reflects the many aspects of fantasizing activity yet to be fully clarified. This may also stem from limited reflective ability in clinical and non-clinical samples regarding mental activity when using self-report assessment tools. Future studies should gather additional information concerning daydreaming content and functions (Brenner et al., 2022; Wen et al., 2024) to attempt to capture the uniqueness of MD even further. Second, although the study groups reached our goal of a minimum of 30 people each, and the overall sample size (*N* = 156) was more than adequate considering the clinical nature of the study and considering that all participants underwent structured clinical interviews, still, sample size for each of the groups was not very large, limiting generalization. Future studies should attempt to replicate these findings with even larger samples. Since ADHD has multiple manifestations of attention deficiencies (Antshel et al., 2014) perhaps a larger sample could have shed more light on the distribution of ADHD persons per their DCQ scores. Third, the interviews were carried out mostly by a single interviewer, preventing the assessment of inter-rater reliability. Fourth, the interviewers were not blinded to the online platform from which participants were recruited (MD, ADHD, or general forum), which may have biased their ratings. Fifth, age differed among groups, with MD and Both being somewhat younger. This may be an inherent feature of MD, which was found to be more prevalent in younger adults than older ones (Soffer-Dudek & Theodor-Katz, 2022). The effect could stem from either a developmental effect (e.g., daydreaming declines with age), an effect of life circumstances (e.g., studying compared to working), or a cohort effect (e.g., cultural exposure to influencers on social media with ideal-looking lives promotes maladaptive fantasizing). In any case, this study focused on the conceptual differences between conditions, and we do not expect that age significantly affected results. Moreover, controlling for age statistically did not change the results. Lastly, demographic data regarding sex and racial and ethnic identification was not collected, but rather, only data on gender (pertaining to the participants’ self-identification) and country, to assess participants’ cultural backgrounds. Future studies should also explore biological sex to assess its possible effect on MD, and assess the ethnic identification of participants within specific countries. A related limitation is that the gender distribution in our sample showed an over-representation for women over men, which is not typical to ADHD; this may possibly reflect differences in the propensity of women to engage with research on mental health online. While our statistical investigation did not find any effect of gender on the results, future studies should further examine how gender may affect daydreaming.

## Conclusions

This study has both theoretical and practical implications. The present investigation demonstrates that ADHD is strongly associated with MW, much more than it is with immersive daydreaming, whereas MD is significantly associated with immersive daydreaming much more than it is with MW. This “double dissociation” suggests that ADHD and MD are separate conditions characterized by differing thought patterns. This supports the conceptualization of MD as a distinct clinical condition in which individuals recurrently delve into immersive daydreaming episodes to avoid distress while hindering their attention and diminishing their interest and active engagement in real-life activities. The results are also practically significant for clinicians and researchers, as MD may commonly be diagnosed as ADHD, and in MD studies, ADHD may inflate MD scores. The recently developed DCQ, a measure of immersive daydreaming properties, was differentially associated with MD and contributed to its screening in the face of ADHD. Consequently, in cases where attention difficulties are reported, administering the DCQ alongside the MDS-16 can improve diagnostic accuracy with a dedicated self-report assessment of immersive, vivid, emotional fantasizing. By identifying MD as a stand-alone condition from ADHD, a targeted protocol could be developed, specifically aimed at increasing the ability to resist the daydreaming urge, promoting positive coping styles over emotional avoidance, nurturing a non-fantasy-based positive self-image, and reducing daydreaming activity to a pleasurable pastime that does not replace real-life personal goals. We hope this study will promote such clinical advancements by demonstrating the immersive daydreaming pattern in MD which diverges from typical ADHD.

## Declaration of competing interest

The authors have declared no conflicts of interest to disclose. Part of this research was supported by the 10.13039/501100003977Israel Science Foundation (grant No. 1444/22), granted to Professor Nirit Soffer-Dudek.

## Footnotes

1 In all analyses conducted, controlling for gender had no significant effect on the results, so we report results without it.

2 To ensure the results reflect a genuine difference between MD and non-MD participants in immersive daydreaming, an ANOVA with multi-comparisons and Tukey correction rather than planned contrasts was also carried out. No change was detected: IDD was higher in the MD and the “Both” groups separately compared to the control and ADHD groups, and did not differ significantly between the two MD groups.

3 The ROC analysis focusing on ADHD-only versus Both pointed at 55 as the optimal cutoff score in the context of an inattentive sample. Although it did improve specificity to 86.8%, it also resulted in a compromised sensitivity of only 61.8% correctly identified MD cases.
